# The effect of caffeine on cutaneous postocclusive reactive hyperaemia

**DOI:** 10.1371/journal.pone.0214919

**Published:** 2019-04-08

**Authors:** Ziva Melik, Tanja Princi, Vittorio Grill, Ksenija Cankar

**Affiliations:** 1 University of Ljubljana, Faculty of Medicine, Institute of Physiology, Ljubljana, Slovenia; 2 University of Trieste, Department of Life Sciences, Trieste, Italy; University of Illinois at Urbana-Champaign, UNITED STATES

## Abstract

**Background:**

Caffeine is reported to be the most widely used pharmacologically active substance. It causes mental stimulation and increases blood pressure. Acute systolic and diastolic blood pressure response to caffeine attenuates in the course of regular caffeine use; tolerance to cardiovascular responses develops in some people. For some hypertension-prone people coffee ingestion may be harmful, and for others it may be beneficial. The aim of our work was to evaluate the effect of caffeine on postocclusive reactive hyperaemia (PORH), a test of microvascular function, and at the same time to monitor the central effects of caffeine on blood pressure and heart rate.

**Methods:**

Heart rate, arterial pressure, and cutaneous laser-Doppler (LD) flux were monitored in 32 healthy volunteers (aged 25.2 ± 4.3 years) before and after they ingested 200 mg of caffeine. LD flux was measured on a finger at rest and after the release of an 8-minute occlusion of digital arteries above the place of LD flux measurement. All parameters obtained after the ingestion of caffeine were compared to the values obtained before caffeine and to the values obtained after a placebo.

**Results:**

We found slightly increased arterial pressure as well as decreased heart rate and resting LD flux (Dunnett’s test, p<0.05) after the ingestion of caffeine. Caffeine significantly reduced the PORH response (Dunnett’s test, p<0.01). The power of the low-frequency oscillations (0.06–0.15 Hz) of LD flux, representing vascular myogenic activity, increased significantly after the ingestion of caffeine at rest and during the PORH response. A correlation was found between the number of cups of coffee regularly consumed and resting LD flux values (R = 0.492, p = 0.00422), peak LD flux values during PORH (R = 0.458, p = 0.00847), and the PORH area (R = 0.506, p = 0.00313) after caffeine consumption.

**Conclusions:**

From the results, we can conclude that caffeine affects cutaneous microvascular function during rest and during a PORH response, and that it increases blood pressure and decreases heart rate.

## Introduction

Caffeine is reported to be the most widely used pharmacologically active substance in the world and is found in a variety of foods, beverages, and medicinal preparations. There are many studies on the cardiovascular effects of acute caffeine intake in humans, but the results are controversial. Caffeine causes mental stimulation and increases blood pressure. The increase in blood pressure in males is mainly due to its effect on systemic vascular resistance [1–3]. The acute blood pressure response to caffeine is attenuated by regular caffeine use; in some people, even tolerance to cardiovascular responses develops. However, regular coffee ingestion may be harmful to some hypertension-prone people [4]. Some researchers report that caffeine increases heart rate variability (HRV) as a consequence of enhanced parasympathetic activity along with a concomitant reduction in sympathetic activity [1,5]. In contrast, other studies conclude that it can have the opposite effect on HRV [3] or that modest amounts of caffeine were found to have neither negative nor positive effects in young, healthy, habitual caffeine consumers [3,6].

Caffeine is a member of the group of methylxanthines, chemicals that have diverse mechanisms of action, including adenosine receptor antagonism, inhibition of cyclic nucleotide phosphodiesterases and mobilization of intracellular calcium through ryanodine receptors [7–9]. Caffeine is generally believed to act only on the adenosine A1 and A2A receptors in the concentration range reached after regular coffee consumption [10]. Several studies argue that the most important mechanism of caffeine action on the cardiovascular system in vivo in humans is adenosine receptor antagonism [11,12].

There are not enough data on the effect of caffeine on microcirculation in humans. Studying cutaneous microvascular reactivity is noninvasive and harmless. Various vasoreactivity tests have been used to study cutaneous microvascular reactivity. To detect overall changes in microvascular function, we used a test of postocclusive reactive hyperaemia (PORH) [13]. PORH is considered to be a locally mediated rise in muscle and skin blood flow that can be measured after the release of an arterial occlusion. The detailed mechanism of PORH remains inadequately explained, but factors that contribute to PORH are numerous: myogenic [14], flow-dependent and local vasoactive substances, e.g. prostaglandins, nitric oxide (NO), adenosine, endothelium-derived hyperpolarizing factor (EDHF), and local concentration of potassium [15]. A number of studies have been focused on the possibility that coffee may increase the risk of heart disease. The results were inconsistent. Since some recent epidemiological studies have demonstrated that coffee drinking is associated with reduced mortality due to cardiovascular disease [16–20], it would be interesting to clarify the detailed mechanism of caffeine action on microvessels during PORH, which is at least partially dependent on adenosine. To our knowledge, there have been only two studies testing the influence of acute caffeine consumption on the cutaneous microvascular response to PORH in humans. The results of the study by Noguchi and colleagues are interesting and in part unexpected [21]. Caffeine elevated blood pressure and decreased finger blood flow and at the same time enhanced the PORH of finger blood flow. In contrast, the study by Tesselaar and co-workers reported no change in blood pressure, heart rate, or PORH [22].

The aim of the present study was to evaluate the effect of a single dose of caffeine on the PORH response, a test of microvascular function, and at the same time to determine the central effects of caffeine on blood pressure and heart rate in young, healthy volunteers.

## Subjects and methods

### Subjects

Thirty-two, young, healthy volunteers (14 males and 18 females) with a mean age of 25.2 ± 4.2 years and a mean body mass index of 21.7 ± 2.2 were recruited for the present study. None of the subjects used any medication or had a history of any diseases that involve even mild Raynaud’s phenomenon. The National Ethics Committee approved the study, and informed consent was obtained from each subject.

### Methods

The subjects were asked not to drink coffee or tea for at least 7 days before the experiment. Three of the subjects were smokers. They were not allowed to smoke at least 8 hours before the experiment. All subjects who did not follow the protocol were excluded from the study. The measurements were performed at room temperature kept between 23 and 25°C, 30 minutes after acclimatization. The subjects lay in a supine position and were instructed not to move during the measurements in order to avoid movement artifacts as much as possible.

#### Heart rate and arterial blood pressure

Heart rate and arterial blood pressure were measured at the forearm by the Riva-Rocci method.

#### Cutaneous microvascular blood flow

Cutaneous microvascular blood flow was assessed by Laser-Doppler (LD) fluxmetry. The LD flux was measured with Periflux P4001 Master/4002 Satellite LD monitors (Perimed, Sweden). With this technique, a laser light is used to transilluminate proximally one cubic millimeter of skin tissue, and the Doppler principle is adopted to measure the velocity of the red blood cells in skin microvasculature. The LD flux signal is a stochastic representation of the number of moving cells in the tissue volume multiplied by their velocities. The principle governing the measurement of skin perfusion with this technique has been described elsewhere [23]. The LD probes (PF401) were attached to the nailfold of one index finger. Power spectral analysis of the LD flux was performed by the Fast Fourier Transform method using Nevrokard software (Medistar, Slovenia) for the analysis of the LD flux signal.

### Protocol

**Fig 1:**
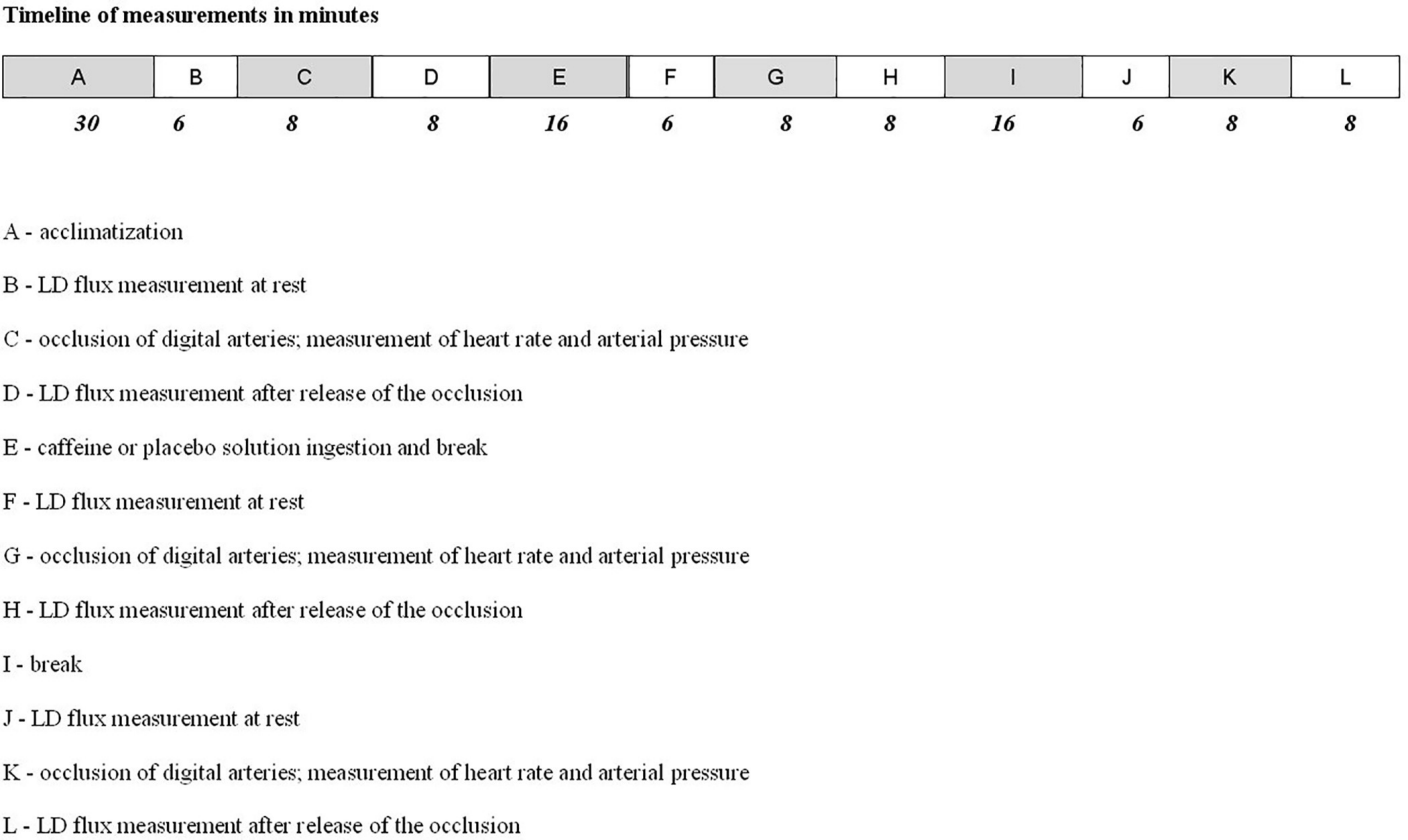
Timeline of the experimental protocol.

**Fig 2:**
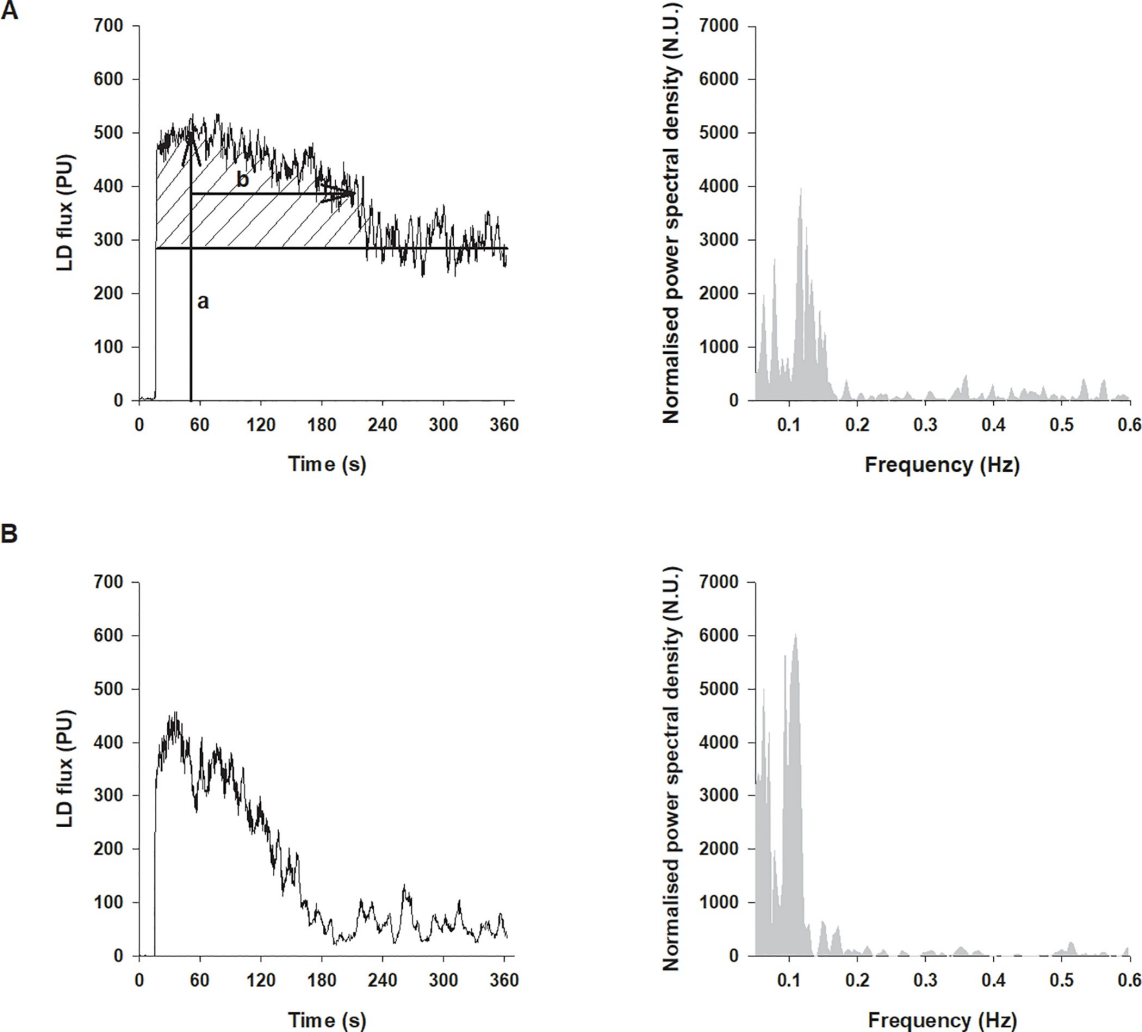
**Typical LD flux PORH response and its power spectral density before (A) and after ingestion of caffeine (B).** Indices of the response measured: peak LD flux (a), T/2 (b), and area under the LD flux curve (dashed area).

Each subject was measured on two separate occasions. The measurement protocol was the same each time with the exception of the ingested substance, which was caffeine on one occasion and a placebo on the other. The timeline of the experimental protocol is presented in Fig 1. LD flux, heart rate, and arterial blood pressure were measured. LD flux was measured at the nailfold. The nailfold was chosen for measurement because it involves only nutritional circulation. Other places accessible for measuring blood flow involve both nutritional and thermoregulatory circulation. The measuring periods were 6 minutes at rest and 6 minutes after the release of an 8-minute digital arteries occlusion (PORH response), i.e., until the LD flux returned to the pre-occlusion value, thus enabling the measurement of the area under the flux curve during the PORH response above resting values (area under the curve), Fig 2. An occlusion period of 8 minutes was chosen because a shorter time would have caused a smaller PORH peak flow, and prolongation of occlusion over 8 minutes would cause no further increase in PORH peak flow [24,25]. A miniature cuff was placed around the proximal phalange and inflated to 300 mmHg to ensure that digital arteries were occluded in all subjects [24,25]. Measurements were repeated 30 and 60 minutes after the ingestion of 200 mg of caffeine dissolved in 100 mL of water or 100 mL of a placebo solution. Values obtained before ingestion were used as the reference values. We calculated the following indices of the PORH response: the mean LD flux before the onset of arterial occlusion (pre-occlusive LD flux value), the peak LD flux value during hyperaemia, the time elapsed to the peak value (time-to-peak), the time from arterial occlusion release to the return of LD flux to its pre-occlusive value (PORH duration), the time for LD flux to decline to 50% of its peak response value (half recovery time—T/2), and the area under the flux curve during the PORH response above resting values (area under the curve) [13, 25,26]. In accord with the studies by Noguchy and co-workers [21] and Yvonne-Tee and co-workers [26], the relative reactive hyperaemia value was calculated thus: 100x(peak LD flux–resting LD flux)/resting LD flux.

### Statistics

Heart rate, systolic and diastolic arterial pressure, and mean resting LD flux values obtained 30 and 60 min after caffeine or placebo ingestion were compared to values obtained before ingestion by a one-way repeated measures ANOVA (Dunnett’s test). We also compared the values of PORH indices before and after the ingestion of caffeine or placebo solution by a one-way repeated measures ANOVA (Dunnett’s test).

The correlations between the quantity of coffee regularly consumed by each subject and each of the factors—heart rate, systolic and diastolic arterial pressure, resting LD flux, and PORH parameters in response to caffeine ingestion—were determined by linear regression (Pearson correlation coefficient test).

For a spectral analysis of the LD flux signal, the Fast Fourier transform (Hanning window; normalized power spectral density) was used. The 360 seconds recording was sampled at 500 samples/s and then downsampled to a total of 512 samples (points). In accord with the research of Stefanovska and co-workers (1999), oscillations of the LD flux signal were divided into five frequency bands: ultra-low, 0.0095–0.02 Hz, endothelium-related metabolic frequency band; very-low, 0.02–0.06 Hz, frequency interval of neural activity; low, 0.06–0.15 Hz, representing vascular myogenic activity; high frequency, 0.15–0.4 Hz, representing respiration; and very-high frequency; 0.4–1.6 Hz, representing heart rate [27,28]. Since PORH responses are relatively short, recordings longer than 360 seconds were not appropriate. Thus, the ultra-low frequency band is still represented in the spectrum, although not very accurately. The very-high frequency band, which corresponds to heart rate, was determined separately.

The term “normalized power spectral density” was calculated as:

absolute power of given component/[sum of absolute powers of all components-F*] x 100

F* = adjustable low frequency limit for n.u. calculation. All results are expressed in mean values and standard errors of the means (SE).

## Results

### Resting values

Compared to resting values, i.e., those before caffeine ingestion, increases in systolic and diastolic arterial pressure as well as decreases in heart rate and resting LD flux were statistically significantly at both 30 and 60 minutes after caffeine ingestion (Dunnett’s test, p<0.01) (Table 1). In contrast, heart rate, arterial pressure and resting LD flux did not change after ingestion of the placebo solution.

**Table 1:** Heart rate, arterial pressure and LD flux values before and after caffeine ingestion at 30- and 60-minute intervals.

	Before caffeine	30 minutes	60 minutes
Heart rate (beats/min)	62.8 ± 1.3	59.9 ± 1.2*	61.3 ± 1.5*
Systolic pressure (mmHg)	109.4 ± 1.3	113.1 ± 1.5*	114.8 ± 1.5*
Diastolic pressure (mmHg)	64.5 ± 1.0	69.2 ± 1.1*	70.1 ± 1.3*
Resting LD flux (PU)	149.1 ± 24.6	80.0 ± 13.3*	71.0 ± 10.8*

Data are shown as means ± SE.

(*-statistically significant difference to the control value before caffeine ingestion at p<0.01).

### The PORH indices

**Fig 3:**
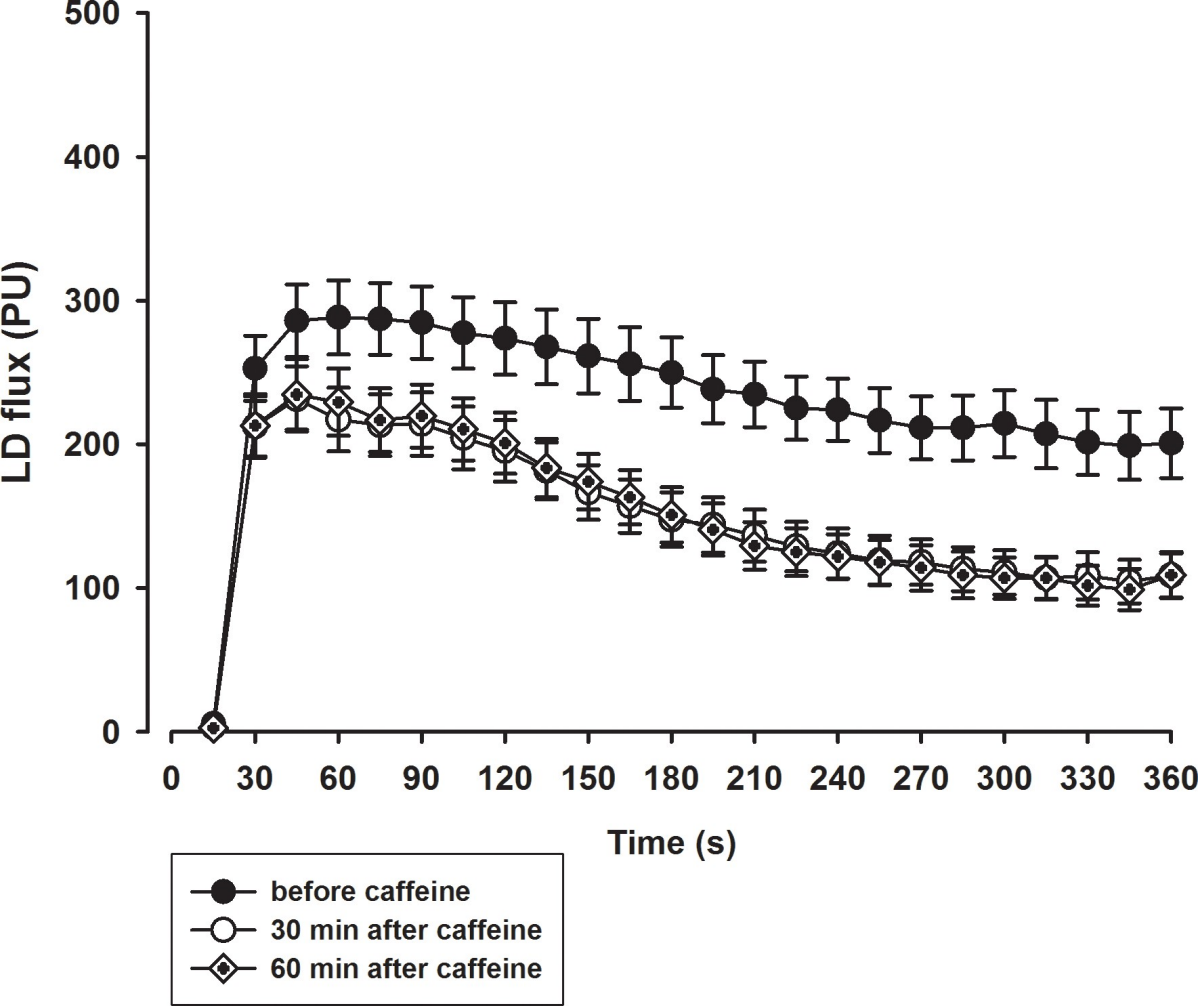
Mean PORH response before ingestion of caffeine and 30 and 60 minutes after ingestion of caffeine.

**Fig 4:**
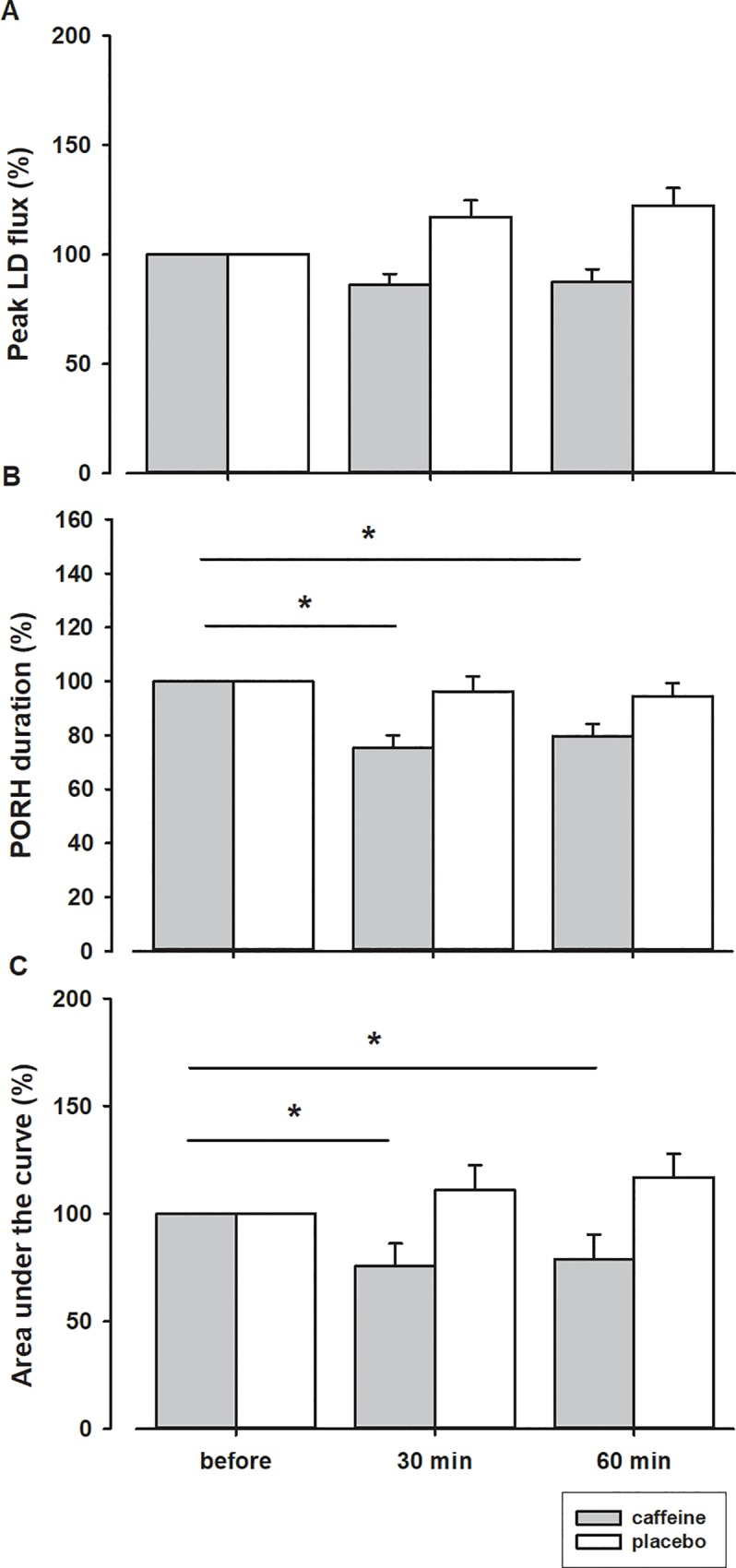
**Peak LD flux (A), duration (B) and area under the LD flux curve (C) after ingestion of caffeine or placebo, expressed as percentage of values obtained before ingestion.** *–statistically significant difference before and after ingestion of caffeine (p < 0.05).

**Fig 5:**
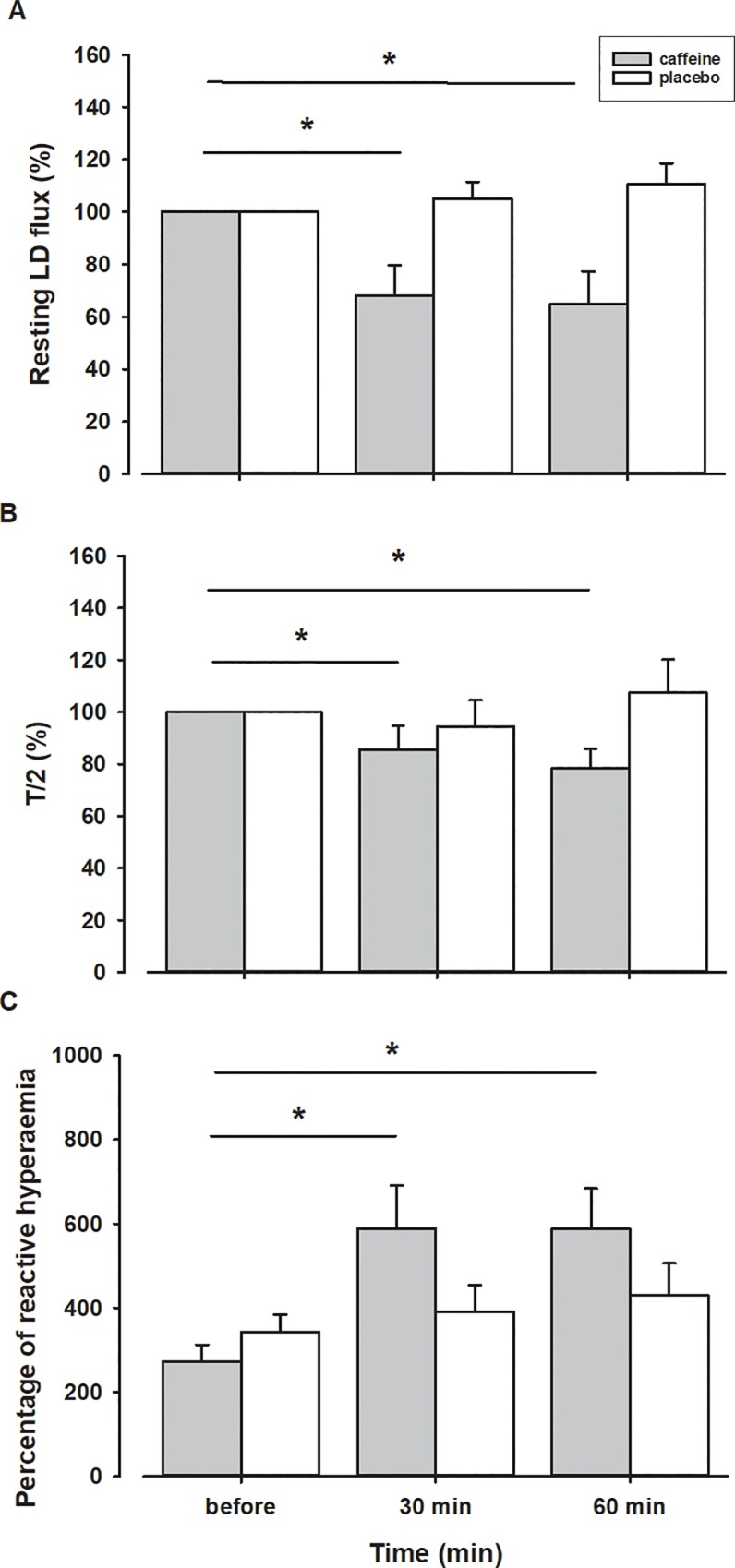
Resting LD flux (A) and half recovery time—T/2 (B) after ingestion of caffeine or placebo expressed as percentage of values obtained before ingestion. Percentage of reactive hyperaemia (C) before and after ingestion of caffeine or placebo. *–statistically significant difference before and after ingestion of caffeine (p < 0.05).

The typical PORH response and power spectral density before and after caffeine ingestion are presented in Fig 2. The mean PORH response 30 and 60 minutes after the ingestion of caffeine was significantly attenuated compared to the response prior to ingestion (Fig 3 and Fig 4). The peak LD flux during PORH (Fig 4A), the PORH duration (Fig 4B), and the area under the LD flux curve (Fig 4C) were significantly reduced after the ingestion of caffeine (Dunnett’s test, p<0.01). In addition, there was a significant decrease in T/2 and an increase in the relative reactive hyperaemia value (Fig 5) but no change in time-to-peak value after the ingestion of caffeine. In contrast, there were no significant differences in obtained values after ingestion of the placebo solution.

### Spectral analysis of LD flux

**Fig 6:**
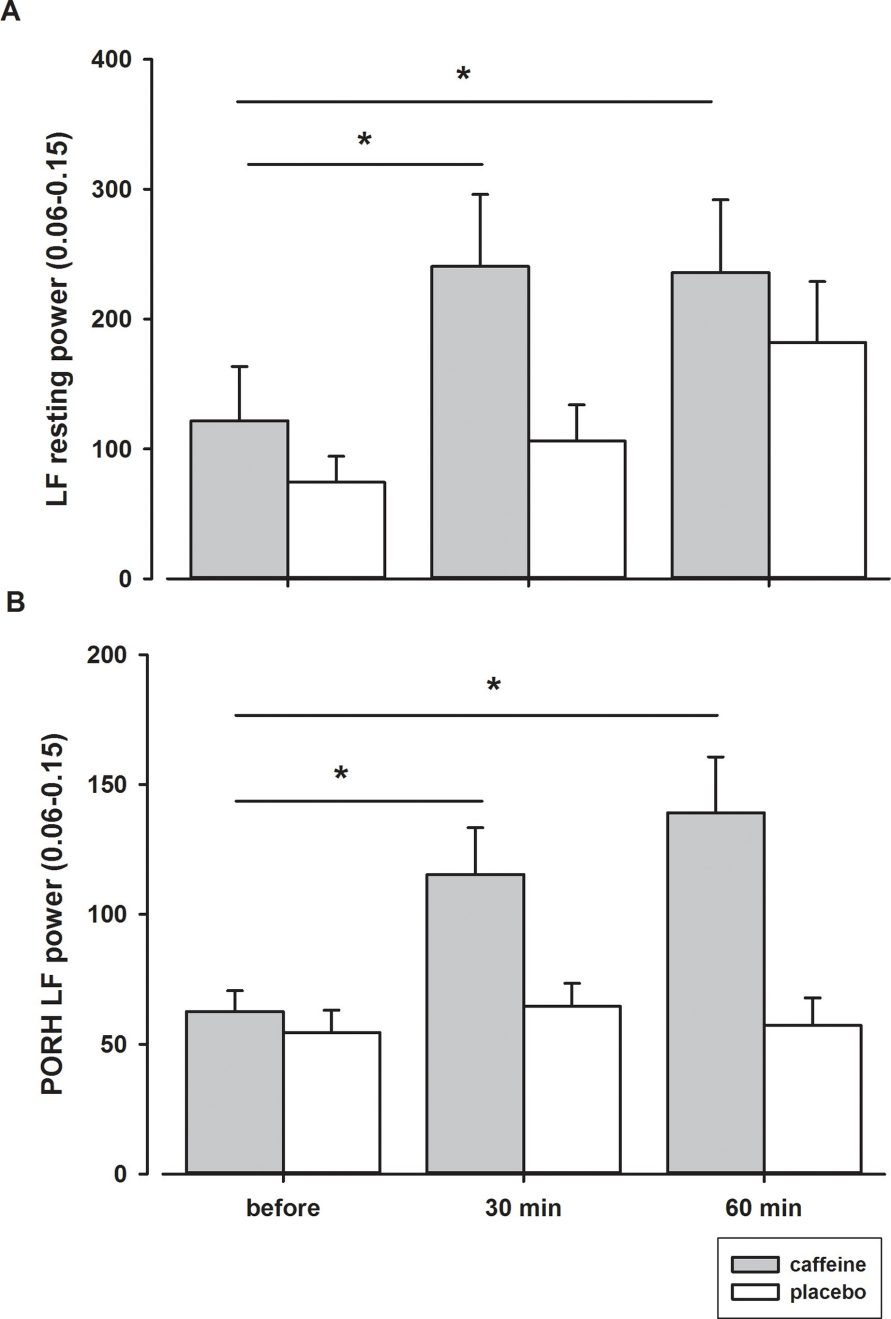
**Power of LF oscillations at rest (A) and during PORH response (B) before and after ingestion of caffeine or placebo.** *–statistically significant difference before and after ingestion of caffeine (p < 0.05).

The power of the low frequency band oscillations increased significantly 30 minutes after the ingestion of caffeine at rest and during the PORH response and remained higher for another 30 minutes (Fig 6 and Table 2). In contrast, the power of the ultra-low frequency, very-low frequency, and high frequency bands of the spectrum did not change after the ingestion of caffeine.

**Table 2:** Spectral analysis of LD signal during PORH response.

	ULF power 0.0095–0.02 Hz [n.u.]	VLF power 0.02–0.06 Hz [n.u.]	LF power 0.06–0.15 Hz [n.u.]	HF power 0.15–0.6 Hz [n.u.]
Before ingestion	24.5 ± 3.4	22.3 ± 2.2	19.2 ± 2.0	11.4 ± 0.8
30 min after caffeine	26.6 ± 4.2	25.6 ± 2.6	27.0 ± 3.7*	11.0 ± 1.5
60 min after caffeine	20.9 ± 3.2	23.4 ± 1.9	28.8 ± 3.0*	13.1 ± 1.8

ULF–ultra-low frequency; VLF–very-low frequency; LF–low frequency; HF–high frequency; n.u.–normalized units

*–statistically significant difference before and after ingestion of caffeine (p < 0.05).

Data are expressed as means ± SE.

### Correlations

A correlation between the PORH area decrease and the heart rate decrease was observed 30 min (R = 0.415, p = 0.0181) and 60 min (R = 0.494, p = 0.0041) after caffeine consumption. There was also a correlation between the number of cups of coffee regularly consumed by test subjects and individual resting LD flux values (R = 0.492, p = 0.00422), peak LD flux values during PORH (R = 0.458, p = 0.00847), and PORH area (R = 0.506, p = 0.00313) after caffeine consumption. In contrast, there were no correlations between the number of cups of coffee regularly consumed and changes in blood pressure or heart rate after caffeine consumption.

## Discussion

The main findings of the present study are that ingestion of a modest amount of caffeine reduces resting cutaneous LD flux and increases low frequency oscillations of postocclusive LD flux. Caffeine also affects the cutaneous microvascular PORH response by diminishing peak LD flux, PORH duration, and consequently the area under the PORH flux curve. To our knowledge this is the first study measuring the direct effect of caffeine ingestion on microcirculation. Two other studies measuring the microcirculatory response to caffeinated or decaffeinated coffee were performed differently and hence are not comparable to ours.

### The effect of caffeine on blood pressure and heart rate

The effect of caffeine on blood pressure was a slight but significant increase in systolic and diastolic blood pressure due to the direct effects of caffeine on vascular tone, on myocardial contractility and conduction, and on the autonomic (sympathetic and parasympathetic) nervous system. However, heart rate decreased. These results were as expected [2,29–31], although in many studies heart rate did not change [21,22,32–34] or even increased [35]. Studies differ in doses of caffeine, time interval after caffeine ingestion, time after the most recent meal, and the number of participants.

The cardiovascular effects of caffeine in concentrations ordinarily achieved after drinking coffee are primarily due to the antagonism of adenosine A(1) and A(2A) receptors. The inhibition of phosphodiesterases or the mobilization of intracellular calcium requires a much higher concentration of caffeine [36].

### The effect of caffeine on PORH

In our study, the PORH response (peak LD flux, PORH duration, T/2, and the area under the PORH LD flux curve) after caffeine ingestion decreased. There are many factors involved in the skin PORH response: the accumulation of vasodilator metabolites (e.g. adenosine); substances released from the endothelium in response to increased shear stress (prostaglandins, EDHF, NO); vascular smooth muscle relaxation in response to reduced transmural pressure in resistance vessels distal to the site of circulatory arrest [15,37,38]. Caffeine, through the antagonism of adenosine receptors, decreased the PORH response.

The study by Noguchi and co-workers [21] presented the “percentage of reactive hyperaemia”, calculated as 100x(peak hyperaemic flow–resting flow)/resting flow. In their and in our study, the so-calculated “relative reactive hyperaemia” increased after caffeine ingestion.

In contrast to the results of the present study, where caffeine reduced the PORH response after the release of the digital arteries occlusion, the study of Tesselaar and co-workers showed that caffeinated coffee did not affect the PORH response after 5-minutes of forearm arterial occlusion [22]. The absence of the effect might be due to the different mechanisms involved in skin PORH after a release of a digital arteries occlusion compared to the PORH response after the release of a forearm occlusion. The postocclusive increase of shear stress releases more NO in the large, conductive arteries and more EDHF in the small, resistance vessels. In addition, a significant dependence of peak flow amplitude on arterial pressure and time of occlusion were reported [24]. Therefore, the mechanisms involved in the PORH response after an occlusion of the brachial artery differ from that of digital arteries. The methodology of the study by Noguchi and co-workers as well as that by Tesselaar and co-workers differs from ours. Time and place of occlusion, quantity and form of caffeine ingestion, and number of participants are different. Therefore, the results of these studies do not exclude one another but are complementary.

The postocclusive reactive hyperaemic response after the release of a digital arteries occlusion consists of two independent parts: 1) peak flow, reflecting a sudden increase of blood flow in the skin microvessels after the removal of the occlusion (an indicator of the increased shear stress) [39,40] and 2) a more prolonged hyperaemic phase, reflecting metabolic debt repayment to the tissue [41]. This second part of PORH, consisting of vasomotion of myogenic origin, independently affects the post-peak-flow re-perfusion and can increase mean blood flow up to 40–60% [42]. An increase in blood flow due to oscillations in the arteriolar diameter depends on the initial smooth muscle cell tone [42]. Additionally, blood flow distribution at bifurcations depends on the oscillation waveforms [42,43].

Our results also confirm that regular caffeine consumption leads to lessened difference in the resting LD flux and the PORH response before and after ingestion of a single dose of caffeine, as represented by the correlation between the amount of regular caffeine consumption and the PORH response to the single dose of caffeine.

### The effect of caffeine on vasomotion

In our study the overall effect of caffeine on the cutaneous vessel wall was vasoconstriction, indicated by elevated blood pressure and decreased resting cutaneous LD flow. At the same time, the power of the low-frequency interval, 0.06–0.15 Hz, of PORH oscillations increased after caffeine consumption.

Microvascular vasomotion is defined as the spontaneous rhythmic contraction and dilatation of the smooth muscles in the walls of small blood vessels [43,44]. There is some evidence indicating that vasomotion may be involved in the regulation of microvascular blood flow distribution and oxygen delivery to tissues [45,46] and two theoretical modeling studies [47,48] suggest that vasomotion may improve oxygen delivery in some cases.

Caffeine acts on the smooth muscular cells through different mechanisms with an opposite effect. Through direct action caffeine activates the ryanodine channels in the endoplasmic reticulum, activates the nonselective channel for cations, inhibits the cAMP phosphodiesterase, inhibits the inositol trisphosphate receptor, inhibits myosin light chain kinase, increases Ca2+ concentration in a region distant from the contractile apparatus, inhibits voltage-dependent Ca2+ channels, and blocks adenosine receptors. Through indirect action, caffeine increases the production of nitric oxide, increases the production of renin, and stimulates the sympathetic system [49,50]. It seems that in our experimental setting the mechanisms that cause an increase in vascular smooth muscle activity prevailed.

### Study limitations

For the spectral analysis of the LD flux signal we used 6-minute recordings. Due to the short length of the recordings, we cannot be certain that the ultra-low frequency oscillations after caffeine did not change.

Both habitual and nonhabitual coffee drinkers were among the subjects in the present study. Some of the nonhabitual coffee drinkers reported concern about consuming 200 mg of caffeine. Nevertheless, all participants in the study consumed 200 mg of caffeine. However, given the concern reported by some nonhabitual coffee drinkers, we cannot be certain that those participants were calm after the caffeine ingestion. This fact could have influenced individual resting LD flux values. However, since resting LD flux values and heart rate decreased after caffeine consumption, we believe that any resultant anxiety had only a minor effect on the results obtained in the present study.

## Conclusions

The results of our study demonstrate for the first time that caffeine affects cutaneous microcirculation by increasing low frequency oscillations of resting and postocclusive LD flux, which is related to spontaneous myogenic activity. The impact on the PORH response is the diminishment of peak LD flux, PORH duration, and consequently the area under the PORH flux curve. In addition, the results confirm that the ingestion of a modest amount of caffeine increases blood pressure, while at the same time reduces heart rate and resting cutaneous LD flux in young, healthy individuals.

## Supporting information

S1 Table (XLSX)
